# Outlook of IL-6 signaling blockade for COVID-19 pneumonia

**DOI:** 10.1186/s41232-020-00134-7

**Published:** 2020-10-05

**Authors:** Misato Hashizume

**Affiliations:** Chugai Pharmaceutical Co., Ltd., 1-1 Nihonbashi-Muromachi 2-Chome, Chuo-ku, Tokyo, 103-8324 Japan

**Keywords:** Interleukin-6, COVID-19 pneumonia, Cytokine release syndrome, Tocilizumab, Sarilumab, Siltuximab

## Abstract

In this review article, it is highlighted the implications of pleiotropic functions of interleukin-6 (IL-6) for one of the therapeutic options targeting for COVID-19. Moreover, it is discussed how real-world data and trials with IL-6 signaling blockade will be crucial in informing the development of new treatment for COVID-19 pneumonia.

Given physiological roles of IL-6 in inflammatory conditions and the data from real world, IL-6 signal inhibitors, along with standard of care (SOC) treatment, might provide efficacy, offering the potential to treat COVID-19 in hospitalized populations more effectively than current SOC alone. Therefore, on-going and planned randomized placebo-controlled studies in combination with SOC and other therapeutics to assess safety and efficacy of IL-6 signal inhibitors in hospitalized patients with severe COVID-19 pneumonia will be warranted to address the high unmet need and burden of disease in this severely ill population.

## Background

In this review article, it is highlighted that the implications of physiological role of interleukin-6 (IL-6), for one of the therapeutic options targeting for COVID-19 which is the acronym of “coronavirus disease 2019,” are caused by a novel coronavirus strain (severe acute respiratory syndrome, SARS-CoV-2). Moreover, it is discussed how real-world data and trials with IL-6 signaling blockade will be crucial in informing the development of new treatment for COVID-19 pneumonia.

## IL-6 and its therapeutic target

**Fig. 1:**
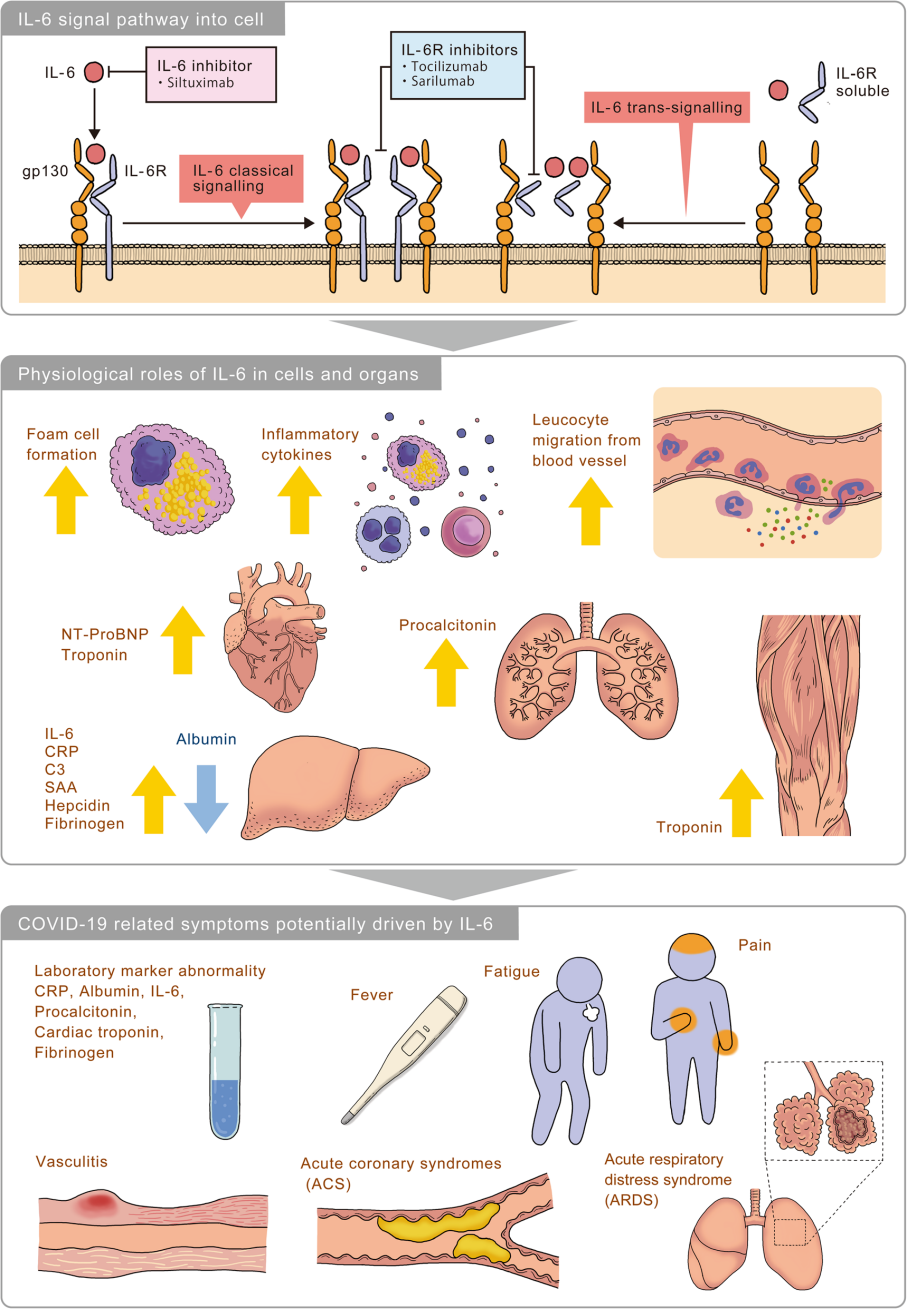
IL-6 involvement in COVID-19- key manifestations and hypothetical mechanisms. IL-6 binds to IL-6 receptor (IL-6R) and glycoprotein 130 (gp130) to form a hexametric complex which is associated with the classical and trans-signaling pathways. Pharmacological inhibitors of IL-6 signaling prevent IL-6 from binding to IL-6R by targeting either the cytokine itself or the receptor. IL-6 is well known to play various physiological roles in cells and organs. For example, in macrophage IL-6 induces foam cell formation [5] and in macrophage, B cell, T cell and neutrophil, IL-6 induces inflammatory cytokines [3, 4]. And IL-6 induces leucocyte migration from blood to organs [3, 6]. IL-6 controls the level of NT-ProBNP and troponin [7]. In lung, IL-6 induces procalcitonin [8]. In liver, acute phase proteins including IL-6, CRP, C3, SAA, hepcidin, and fibrinogen are upregulated by IL-6, and albumin is downregulated by IL-6 [3, 4]

IL-6 was found in 1973 as a soluble factor that is secreted by T cells and is important for antibody production by B cells [1]. Since its discovery more than 40 years ago, the IL-6 pathway has emerged as a pivotal pathway involved in immune regulation in health and dysregulation in many diseases [2–4]. IL-6 is a pleiotropic pro-inflammatory multifunctional cytokine produced by a variety of cell types and has been shown to be involved in diverse physiological processes such as T cell activation; induction of acute phase proteins; stimulation of hematopoietic precursor cell growth and differentiation; proliferation of hepatic, dermal, and neural cells; bone metabolism; lipid metabolism; atherosclerosis; hepatoprotection; and fibrosis as shown in Fig. 1 [2–4]. Elevated tissue and serum levels of IL-6 have been implicated in the disease pathology of several inflammatory and autoimmune disorders including multiple myeloma, Crohn’s disease, rheumatoid arthritis (RA), Castleman disease, systemic juvenile idiopathic arthritis (sJIA), polyarticular juvenile idiopathic arthritis (pJIA), adult-onset Still’s disease (AOSD), ankylosing spondylitis, psoriatic arthritis, systemic lupus erythematosus, giant cell arteritis (GCA), Takayasu arteritis (TAK), systemic sclerosis, and cytokine-release syndrome (CRS), and targeting of the IL-6 pathway has led to innovative therapeutic approaches for various rheumatic conditions such as RA, JIA, AOSD, GCA, TAK, and others such as Castleman disease or chimeric antigen receptor (CAR) T cell-induced CRS [2].

## Tocilizumab in cytokine-release syndrome of CAR T therapy

CRS has been identified as a clinically significant, on-target, off-tumor side effect of the CAR T cell therapies used for treatment of malignancies. Characteristics of CRS include fever, headache, encephalopathy, hypotension, tachycardia, capillary leak, and multi-organ dysfunction [9]. The reported incidence of CRS after CAR T cell therapy ranges from 37 to 93%, with 1 to 46% of patients experiencing the severe or life-threatening form [9]. Serum levels of inflammatory cytokines are elevated, particularly IL-6 [9]. The severity of symptoms may correlate with the serum cytokine concentrations and the duration of exposure to the inflammatory cytokines [9].

On August 30, 2017, the U.S. Food and Drug Administration approved tocilizumab for the treatment of severe or life-threatening CAR T cell-induced CRS in adults and in pediatric patients 2 years of age and older [10], and tocilizumab is also approved for CAR T-induced severe or life-threatening CRS in the EU and certain other countries [11]. The approval of tocilizumab was based on a retrospective analysis of data for patients treated with tocilizumab who developed CRS after treatment with tisagenlecleucel or axicabtagene ciloleucel in prospective clinical trials [12].

## Hypothesis of physiological role of IL-6 in COVID-19

Though there are many things that have not yet revealed, COVID-19 shows acute respiratory illness with fever, loss of smell and taste, and respiratory symptoms, such as cough and shortness of breath as common symptoms caused by SARS-CoV-2 [13]. A striking feature of severe COVID-19 is the rapid progression of respiratory failure which is acute respiratory distress syndrome (ARDS) soon after the onset of dyspnea and hypoxemia due to lymphocyte infiltration into interstitial and alveolar spaces [14, 15]. Severe COVID-19 could also lead to acute coronary syndromes (ACS), kidney, and liver injury, in addition to vasculitis, coagulopathy, and shock [16–21]. These organ failures may be associated with a CRS characterized by high fevers, thrombocytopenia, hyperferritinemia, and elevation of other inflammatory markers including C-reactive protein (CRP).

Unraveling the therapeutic potential of IL-6 signal inhibitors for COVID-19 is a matter of their mode of action corroborated by data of IL-6 signal inhibitors in various diseases since COVID-19 has similar symptoms as inflammatory and autoimmune diseases where IL-6 signal inhibitors have already shown. For example, in the randomized double-blind placebo-controlled study (RCT/PBO) for sJIA, tocilizumab significantly improved systemic features including fever and rash and laboratory features including CRP, anemia thrombocytosis, and hyperferritinemia [22]. In RCT/PBO for RA, tocilizumab and sarilumab significantly improved fatigue, pain, and laboratory features [23, 24]. In RCT/PBO for SSc, regarding the mean change in forced vital capacity which was the secondary endpoint, tocilizumab performed better than placebo, suggesting a potentially clinically important effect of tocilizumab on the preservation of lung function [25, 26]. In RCT/PBO for GCA, over half of patients treated with tocilizumab could achieve sustained remission without glucocorticoid which is free from vasculitis-related symptoms including pain, vision loss, and stroke [27]. For ACS, in RCT/PBO, tocilizumab significantly reduced high-sensitivity CRP and high-sensitivity troponin T, encouraging further trials to assess the potential effects of IL-6 inhibition on clinical outcomes in ACS [28].

## Elevation of IL-6 in COVID-19

In inflammatory condition, IL-6 is well-known to elevate, implicating that CRP which is one of the surrogate markers of IL-6 elevation allows us to detect that IL-6-mediated inflammation occurs in our body [4]. Since IL-6 is released by immune cells including macrophage and T cells, they are activated by virus or bacteria or other immune cells [2–4]. IL-6 acts like a messenger to activate other immune cells to fight the infection [3, 4]. Indeed, in patients with COVID-19, it has been reported that IL-6 level is significantly elevated and associated with adverse clinical outcomes including severity and mortality of COVID-19 in published papers and pre-print papers [29–31]. Based on these facts, IL-6 or CRP, which is its surrogate marker can support physicians, together with other tests and vital signs, to decide if intensive care therapy is necessary, or if a specific therapy (e.g. mechanical ventilation) should be started or intensified and how long intensive care therapy is needed. In the USA, FDA declared the emergency use authorization for use of IL-6 immunoassay for COVID-19 patients [32]. The assay is used to assist in identifying severe inflammatory response in patients with confirmed COVID-19 illness to aid in determining the risk of intubation with mechanical ventilation, in conjunction with clinical findings and the results of other laboratory testing [32]. And, in the Chinese Clinical Guidance for COVID-19 Pneumonia Diagnosis and Treatment (7th Edition), published by China National Health Commission on March 4, 2020, IL-6 is the part of measurements [33]. Moreover, the Italian Society of Infectious and Tropical Diseases (Handbook for the care of people with disease COVID-19, Edition 2.0) mentions that IL-6 is the central mediator of cytokine release syndrome toxicity and IL-6 could help to identify most severe COVID-19 patients who could benefit from further therapy [34].

## REAL world experience with IL-6 blockade in COVID-19 pneumonia

In the beginning of the world pandemic, physicians in China initiated the off-label use of tocilizumab in the treatment of COVID-19 pneumonia. In February 2020, 21 patients with severe or critical COVID-19 pneumonia were treated with tocilizumab 400 mg IV plus standard of care (SOC) [35]. The average age of the patients was 56.8 ± 16.5 years, ranging from 25 to 88 years. Seventeen patients (81.0%) were assessed as severe, and 4 patients (19.0%) were assessed as critical. Most patients (85.0%) were presented with lymphopenia. CRP levels were increased in all 20 patients evaluated (mean 75.06 ± 66.80 mg/L). The median procalcitonin (PCT) value was 0.33 ± 0.78 ng/mL, and only 2 of 20 patients (10.0%) were presented with an abnormal value. Mean IL-6 level before tocilizumab was 132.38 ± 278.54 pg/mL (normal < 7 pg/mL). SOC is consisted of lopinavir, methylprednisolone, other symptom relievers, and oxygen therapy as recommended by the Diagnosis and Treatment Protocol for Novel Coronavirus Pneumonia (Trial Version 6) (China National Health Commission 2020) [36]. All 21 patients had received routine SOC treatment for a week before deteriorating with sustained fever, hypoxemia, and chest computed tomography (CT) image worsening. Eighteen patients (85.7%) received tocilizumab once, and 3 patients (14.3%) had a second dose due to fever within 12 h. According to the authors, after tocilizumab treatment, fever returned to normal, and all other symptoms improved remarkably. Fifteen of the 20 patients (75.0%) had lowered their oxygen intake, and 1 patient needed no oxygen therapy. CT scans showed significant remission of opacities in both lungs in 19 of 21 patients (90.5%) after treatment with tocilizumab. The percentage of lymphocytes in peripheral blood, which was decreased in 17 of 20 patients (85%) before treatment (mean 15.52 ± 8.89%), returned to normal in 10 of 19 patients (52.6%) on the fifth day after treatment. Abnormally elevated CRP decreased significantly in 16 of 19 patients (84.2%). No adverse drug reactions and no subsequent pulmonary infections were reported. Nineteen patients (90.5%) were discharged at the time of the report, including 2 critical patients. There were no deaths among the 21 treated patients. The study authors concluded that tocilizumab is an effective treatment for patients with severe COVID-19 pneumonia [35].

CORIMUNO-TOCI which is an investigator initiated study is part of CORIMUNO-19, a French multi-center, open-label, platform study examining the safety and efficacy of immunomodulators in COVID-19 [37]. The study randomized 129 non-ICU patients with moderate-severe pneumonia (requiring supplemental oxygen) 1:1 planned to receive 1 or 2 doses of tocilizumab 8 mg/kg + SOC vs SOC alone. The primary composite outcome was the need for ventilation (non-invasive or mechanical) or death by day 14. As stated in the press release from 27 April regarding this composite endpoint, a significantly lower proportion of patients in the tocilizumab required either ventilation (non-invasive or mechanical) or died by day 14 [37].

TOCIVID-19 is the first and largest study on tocilizumab authorized by the Italian Medicines Agency (AIFA) [38, 39]. The primary analysis concerns 301 patients registered for the phase 2 study (in 20 h between 19 and 20 March), and 920 patients subsequently registered between 20 and 24 March, coming from 185 clinical centers distributed throughout Italy. These patients had all been hospitalized due to a picture of pneumonia that occurred during coronavirus infection, and showed signs of respiratory failure. On the other hand, patients intubated for more than 24 h were excluded from this analysis and will be further investigated. The group of 920 patients was included in the analysis with the aim of confirming the results observed in the phase 2 study. Due to the limited initial availability of the drug, and the very rapid request from the centers, in both groups, only 60% of the patients were treated with tocilizumab, in some cases even at a significant time after registration. Furthermore, probably due to a selection made in the centers, the treated patients were clinically worse than the untreated ones, with more severe respiratory insufficiency and more intensive forms of respiratory assistance. Over the next 30 days, 67 deaths were recorded in the phase 2 study. As defined by the protocol [38], the primary analysis was conducted on the 14 and 30 day lethality rate. In particular, at 14 days, the lethality rate reported in phase 2 was 18.4%, considering all patients, and 15.6%, considering only those who received the drug, both lower, but not statistically significantly, versus 20% expected a priori on the basis of data provided by the Istituto Superiore di Sanità. On the other hand, the results are statistically significant in the 30-day analysis, when the lethality values are 22.4% in all patients and 20.0% in the treated only compared to > 30% expected a priori. The validation group of 920 patients is characterized by a much better prognosis than in phase 2, particularly with regards to patients not treated with tocilizumab. In fact, at 14 days, lethality is 11.4% in all patients and 10.9% in treated patients, and at 30 days, 18.4% in all patients and 20.0% in treated patients. On one hand, these results confirm those reported in phase 2, but on the other, they introduce a necessary element of caution in the interpretation. The analysis of adverse events conducted in the joint population of 708 treated patients did not show relevant signs of specific toxicity, other than the expected adverse events in the underlying pathological condition. The study authors concluded that the TOCIVID-19 study, despite the limitations of a single-arm study, made more complex by the rapid enrollment and the corresponding limited availability of the drug suggests that tocilizumab may significantly reduce mortality to 1 month, but that its impact is less relevant on early mortality [39].

In Italy, another large observational cohort study (TESEO study) was conducted and published by Giovanni G et al. Five hundred forty-four patients had severe COVID-19 pneumonia and were included in this study [40]. Fifty-seven (16%) of 365 patients in the standard care group needed mechanical ventilation, compared with 33 (18%) of 179 patients treated with tocilizumab intravenous and subcutaneous. Seventy-three (20%) patients in the standard care group died, compared with 13 (7%; *p* < 0.0001) patients treated with tocilizumab. After adjustment for sex, age, recruiting centre, duration of symptoms, and SOFA score, tocilizumab treatment was associated with a reduced risk of invasive mechanical ventilation or death (adjusted hazard ratio 0.61, 95% CI 0.40–0.92; *p* = 0.020). Twenty-four (13%) of 179 patients treated with tocilizumab were diagnosed with new infections, versus 14 (4%) of 365 patients treated with standard of care alone (*p* < 0.0001).

Based on these reports, on March 3, 2020, China’s National Health Commission included tocilizumab IV in the 7th updated diagnosis and treatment plan for COVID-19, making it the first of many countries to add tocilizumab in their national treatment guidance [33]. Other countries that have included tocilizumab in COVID-19 treatment guidelines include Italy, Spain, Greece, Switzerland, Ireland, Russia, Poland, Qatar, Lebanon, Egypt, Israel, and Japan.

Since another IL-6 receptor inhibitor, sarilumab is only marketed in subcutaneous formulation, and no reports from real-world data have been presented as far as it was searched as of 2 June 2020.

## On-going and planned industory sponsored trials

Despite the millions of cases and hundreds of thousands of deaths that have occurred in this COVID-19 pandemic, high standard clinical trials such as RCT/PBO should be conducted in order to evaluate efficacy and safety profile of candidate therapeutics. Although it is used to take a while to conduct trials and review documentations for drug application, this urgent task for health authorities and industries all over the world could transform them more agile and cooperative in order to deliver scientifically authorized therapeutics for patients who need treatment. As shown in Table 1, seven industry sponsored trials of two IL-6 receptor inhibitors are on-going and planned for COVID-19 pneumonia patients.

**Table 1:** Overview of industry sponsored clinical trials of IL-6 signal inhibitors for COVID-19 pneumonia

Drug	Trial name	Location	Design	Patient population and number	Primary endpoint
Tocilizumab	COVACTA NCT04320615 [41]	Global	DB/PBO TCZ + SOC vs PBO + SOC	Hospitalized with COVID-19 pneumonia confirmed per a positive PCR of any specimen and evidenced by chest x-ray or CT scanand SpO2 ≤ 93% or PaO2/FiO2 < 300 mmHg 450	Clinical status as per the 7-category ordinal scale at day 28
Tocilizumab	MARIPOSA NCT04363736 [42]	US	OL TCZ 4 mg/kg TCZ 8 mg/kg	Hospitalization with COVID-19 pneumonia confirmed by a positive PCR of any specimen and evidenced by chest x-ray or CT For severe patients, SpO2 ≤ 93% or PaO2/FiO2 < 300 mmHg For moderate patients, CRP > 2 × ULN 100	PK/PD responses to tocilizumab
Tocilizumab	REMDACTA NCT04409262 [43]	Global	DB/PBO TCZ + RDV vs PBO + RDV	Hospitalized with COVID-19 pneumonia confirmed per a positive PCR of any specimen and evidenced by chest x-ray or CT scan and SpO2 ≤ 93% or PaO2/FiO2 < 300 mmHg or requiring supplemental oxygen to maintain SpO2 > 93% 450	Clinical status as per the 7-category ordinal scale at day 28
Tocilizumab	EMPACTA NCT04372186 [44]	US	DB/PBO TCZ + SOC vs PBO + SOC	Hospitalized with COVID-19 pneumonia confirmed per a positive PCR of any specimen and evidenced by chest X-ray or CT scan Requiring more than 6 L/min supplemental oxygen to maintain SpO2 > 93% 379379	Cumulative proportion of participants requiring mechanical ventilation by day 28
Tocilizumab	JapicCTI-205270 [45]	Japan	OL	Hospitalized with COVID-19 pneumonia confirmed per a positive PCR of any specimen and evidenced by chest x-ray or CT scan and SpO2 ≤ 93% or PaO2/FiO2 < 300 mmHg 10+	Clinical status as per the 7-category ordinal scale at Day 28
Sarilumab	NCT04315298 [46]	US	DB/PBO SARI dose1 + SOC vs SARI dose2 + SOC vs PBO + SOC dose3 + SOC vs PBO + SOC	P2: adult patients hospitalized with COVID-19 regardless of disease severity strata P3 cohort 1: adult patients hospitalized with critical COVID-19 on mechanical ventilation at baseline P3 cohort 2: adult patients hospitalized with COVID-19 receiving mechanical ventilation at baseline P3 cohort 3: adult patients hospitalized with COVID-19 receiving high-intensity oxygen therapy without mechanical ventilation at baseline. 2500	Phase 2: Percent change in CRP levels by day 4 Phase 3: Proportion of patients with at least 1-point improvement in clinical status using the 7-point ordinal scale up to day 22
Sarilumab	NCT04327388 [47]	Global	DB/PBO SARI dose1 + SOC vs SARI dose2 + SOC vs PBO + SOC	Hospitalized for less than or equal to 7 days with evidence of pneumonia and has one of the following disease categories: severe disease or critical disease laboratory-confirmed SARS-CoV-2 infection 400	Time to improvement of 2 points in clinical status assessment from baseline using the 7-point ordinal scale up to day 29

*CRP* C-reactive protein, *DB/PBO* double-blind placebo-controlled study, *FiO2* fraction of inspired oxygen, *OL* open label, *PaO2* partial pressure of oxygen in arterial blood, *PBO* placebo, *RDV* remdesivir, *SARI* sarilumab, *SOC* standard of care, *SpO2* peripheral capillary oxygen saturation, *TCZ* tocilizumab

On 27 April 2020, Sanofi and Regeneron provided update on U.S. phase 2/3 adaptive designed trial in hospitalized COVID-19 patients [48]. The randomized phase 2 portion of the trial compared intravenously administered sarilumab higher dose (400 mg), sarilumab lower dose (200 mg), and placebo. It assessed 457 hospitalized patients, who were categorized at baseline as having either “severe” illness (28% of patients), “critical” illness (49% of patients), or “multi-system organ dysfunction” (MSOD) (23% of patients). Patients were classified as “severe” if they required oxygen supplementation without mechanical or high-flow oxygenation; or “critical” if they required mechanical ventilation or high-flow oxygenation or required treatment in an intensive care unit. Preliminary analysis of the phase 2 portion of the trial demonstrated that sarilumab rapidly lowered CRP, a key marker of inflammation, meeting the primary endpoint. Baseline levels of IL-6 were elevated across all treatment arms, with higher levels observed in “critical” patients compared to “severe” patients. Additionally, no new safety findings were observed with the use of sarilumab in COVID-19 patients [48].

On 2 July 2020, Sanofi and Regeneron announced the U.S. phase 3 trial (NCT04315298) of sarilumab 400 mg in COVID-19 patients requiring mechanical ventilation which did not meet its primary and key secondary endpoints when sarilumab was added to the best supportive care compared to the best supportive care alone (placebo) [46, 49]. Minor positive trends were observed in the primary pre-specified analysis group (critical patients on sarilumab 400 mg who were mechanically ventilated at baseline) that did not reach statistical significance, and these were countered by negative trends in a subgroup of critical patients who were not mechanically ventilated at baseline. In the primary analysis group, adverse events were experienced by 80% of sarilumab patients and 77% of placebo patients. Serious adverse events that occurred in at least 3% of patients and more frequently among sarilumab patients were multi organ dysfunction syndrome (6% sarilumab, 5% placebo) and hypotension (4% sarilumab, 3% placebo). Based on the results, the U.S.-based trial has been stopped, including in a second cohort of patients who received a higher dose of sarilumab (800 mg). A separate trial outside of the USA in hospitalized patients with severe and critical COVID-19 using a different dosing regimen [47] is ongoing. Although preliminary results were also presented in the press release, the details of results should be reviewed in future publications since the study is on-going.

## Conclusions

There is currently only a drug licensed for the treatment of patients with COVID-19. Given physiological roles of IL-6 in inflammatory conditions and the data from real world outlined above, IL-6 signal inhibitors, along with SOC treatment, could provide efficacy, offering the potential to treat COVID-19 in hospitalized populations more effectively than current SOC alone. Extensive safety data have previously been generated on the use of IL-6 signal inhibitors in other indications. Therefore, on-going and planned placebo-controlled studies in combination with SOC and others to assess safety and efficacy of IL-6 signal inhibitors in hospitalized patients with severe COVID-19 pneumonia will be warranted to address the high unmet need and burden of disease in this severely ill population.

## Data Availability

Not applicable

## Abbreviations

ACS: Acute coronary syndromes
AOSD: Adult-onset Still’s disease
ARDS: Acute respiratory distress syndrome
C3: Complement 3
CAR: Chimeric antigen receptor
CoV: Coronavirus
COVID-19: Coronavirus disease 2019
CRP: C-reactive protein
CRS: Cytokine-release syndrome
CT: Computed tomography
DB/PBO: Double-blind placebo controlled study
FiO2: Fraction of inspired oxygen
GCA: Giant cell arteritis
gp130: Glycoprotein 130
ICU: Intensive care unit
IL-6: Interleukin-6
IL-6R: Interleukin-6 receptor
IV: Intravenous
NT-ProBNP: *N*-terminal pro- B-type natriuretic peptide
OL: Open label
PaO2: Partial pressure of oxygen in arterial blood
PBO: Placebo
RA: Rheumatoid arthritis
RCT/PBO: Randomized double blind placebo controlled study
RDV: Remdesivir
SAA: Serum amyloid A
SARI: Sarilumab
SARS: Severe acute respiratory syndrome
sJIA: Systemic juvenile idiopathic arthritis
SOC: Standard of care
SpO2: Peripheral capillary oxygen saturation
pJIA: Polyarticular juvenile idiopathic arthritis
TAK: Takayasu arteritis
TCZ: Tocilizumab
